# Maternal and perinatal outcomes in pregnant women with COVID-19 in a referral academic center in Iran

**DOI:** 10.18502/ijrm.v21i9.14403

**Published:** 2023-10-30

**Authors:** Paria Boustani, Laleh Eslamian, Ashraf Aleyasin, Ashraf Jamal, Nasim Eshraghi, Marjan Ghaemi

**Affiliations:** ^1^Student Research Committee, School of Medicine, Tehran University of Medical Sciences, Tehran, Iran.; ^2^Department of Obstetrics and Gynecology, Shariati Hospital, Tehran University of Medical Sciences, Tehran, Iran.; ^3^Vali-E-Asr Reproductive Health Research Center, Family Health Research Institute, Tehran University of Medical Sciences, Tehran, Iran.

##  Dear Editor,

Severe acute respiratory syndrome coronavirus 2 caused the coronavirus disease 2019 (COVID-19), which rapidly became a pandemic. A great number of pregnant women around the world were affected by the COVID-19 pandemic. The risk of severe COVID-19 infection may increase during pregnancy due to physiological adaptations and immunological changes. However, no significant difference was observed in a study comparing the complications of pregnant and non-pregnant women of the same age group (1, 2). On the other hand, it seems that some obstetric complications in mothers with COVID-19 are higher than in the normal pregnant population, and based on the results of a systematic review, pre-eclampsia, preterm birth, and stillbirth are higher in pregnancies affected by COVID-19 (3). However, the data on maternal and neonatal complications are still inconclusive due to the small sample size of the studies.

Here we report the complications and mortality rate of a total of 343 pregnant women who were infected with COVID-19 and hospitalized in a tertiary academic center affiliated to the Tehran University of Medical Sciences, Tehran, Iran from March 2020 to August 2022. The mean age of patients was 32.11 ± 5.82 yr (17-50 yr). 29 (8.5%) patients were in the first trimester, 115 (33.5%) were in the second trimester, and 199 (58%) were in the third trimester of pregnancy. The oxygen saturation was < 94% in 103 (30.03%) of the individuals arriving at the emergency department. The most common underlying disease among these women was hypothyroidism in 95 (27.70%) cases, followed by gestational diabetes mellitus in 64 (18.66%), and hypertension in 10 (2.91%) cases.

Participant's median length of stay at the hospital was 6 days (interquartile range: 4-8). The results indicated that 82 (23.91%) had severe COVID-19 requiring admission to the intensive care unit. 14 mothers (4.1%) needed endotracheal intubation, and 3 (0.9%) underwent tracheostomy. The frequency of complications among ICU admitted cases was 8 (9.75%) acute respiratory distress syndrome, 2 (2.44%) pneumothorax, 4 (4.88%) acute kidney injury, 1 (1.22%) deep vein thrombosis, 1 (1.22%) myocarditis, and 10 (12.19%) maternal mortalities. These complications were not seen in non-ICU admitted individuals. There were also 8 (2.33%) cases of pre-eclampsia.

Data on the pregnancy outcomes of 79 pregnant women was available. The results indicated that 10 (12.7%) cases had a vaginal delivery and 69 (87.3%) were delivered via cesarean section. 39 (49.37%) women had preterm labor, and the number of preterm labor was significantly higher in ICU-admitted (21 [7.78%]) than in non-ICU admitted (18 [34.61%]) cases (p < 0.001). Moreover, 7 (8.54%) pregnant women hospitalized in the ICU had fetal demise compared to 5 (1.9%) non-ICU admitted cases (p = 0.01). The 1-min Apgar score of neonates whose mothers were admitted to the ICU (6.84 ± 2.78) was not significantly lower than other neonates (7.7 ± 2.27) (p = 0.142). The maternal mortality rate of total participants was 10 (2.91%). The frequency of stillbirth was 1 (0.29%), and 51 (64.5%) neonates were admitted to the neonatal intensive care unit.

This study indicated that intrauterine fetal demise and preterm birth risk were significantly higher in pregnant women with severe COVID-19 disease requiring ICU admission. Consistent with our findings, previous investigations revealed that COVID-19 infection (especially, in women with severe or critical disease) could lead to several maternal and fetal adverse outcomes, such as pre-eclampsia, preterm birth, and intrauterine fetal demise (4, 5). An increased risk of venous thromboembolism was also reported to be associated with the severity of illness (6). Prophylactic anticoagulant therapy was initiated for all pregnant women in our study; therefore, only one case of venous thromboembolism was reported. Our results showed increased cesarean section rates in this referral center during the COVID-19 pandemic. Recent studies have also shown that a high rate of cesarean delivery is related to the severity of COVID-19 disease (7).

There was an exponential increase in the COVID-19 vaccination rate in August 2021 in Iran. The last case of maternal mortality from COVID-19 in our hospital was reported 2 months later, on October 6 ${}^{\text{th}}$ , 2021 (8-10).

Although most cases were discharged from the hospital without significant morbidity, COVID-19 may lead to major perinatal and maternal complications. Therefore, women with COVID-19 during pregnancy should be evaluated carefully and followed until delivery to reduce adverse outcomes.

##  Conflict of Interest

None.
